# Supplementary material for: Transcriptional Regulation of RUNX1: An Informatics Analysis
Source: Genes (Basel). 2021 Jul 29;12(8):1175. doi: 10.3390/genes12081175 (PMC8395016; doi:10.3390/genes12081175)
Supplement: Supplementary file 1 [file genes-12-01175-s001.zip › genes-1265484-supplementary.pdf]

Supplementary Figure S1

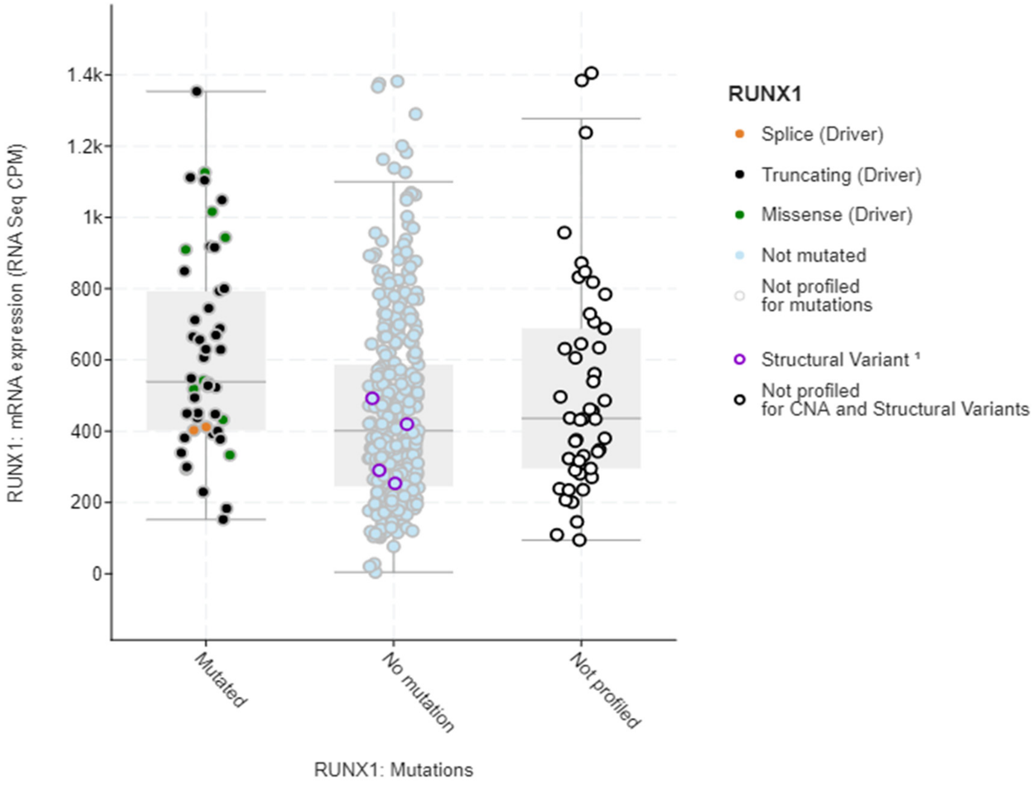

Supplementary Figure S1. Variation in *RUNX1* expression according to *RUNX1* mutation status. The plot represents 451 AML samples in cBioportal with the level of expression of *RUNX1* graphed according to mutation status. 10.6% of samples with mutations in *RUNX1* have altered *RUNX1* expression. 79.2% of AML samples exhibit a wide range of *RUNX1* expression but have no mutations in *RUNX1*. A further 10.2% require mutational profiling, but also have a wide range of *RUNX1* expression.

Supplementary Table S1: Cell types with R1RE RNA expression

Regulatory region	Previous name / location relative to P1 (kb)	Cell type expressing regulatory region RNA
R1RE1	Mouse and Human *Runx1* relative to P1 +23/ +24/ eR1/RE1	Haematopoietic
R1RE2	mouse -371	Skin, Prostate
R1RE3	mouse -368	Not Expressed
R1RE4	mouse -327	Skin, Liver, Brain, Lung, Haematopoietic,Stomach, Oesophagus, Colon, Gall Bladder, Mesenchymal
R1RE5	human -250/ E6	Prostate, Brain, Haematopoietic,Colon, Gall Bladder, Mesenchymal
R1RE6	mouse -181	Not Expressed
R1RE7	human -188/ E4	Not Expressed
R1RE8	human -139/ E1	Liver, Breast, Brain, Lung, Haematopoietic,Oesophagus,
R1RE9	mouse -101	Not Expressed
R1RE10	mouse -58	Not Expressed
R1RE11	mouse -43	Brain, Lung, Haematopoiesis,
R1RE12	human -5	Skin, Brain, Haematopoietic,Oesophagus, Mesenchymal, Kidney
R1RE13	mouse +3	Haematopoietic, Mesenchymal, Kidney
R1RE14	mouse +32	Prostate, Brain, Haematopoietic,Oesophagus, Mesenchymal, Kidney

Regulatory region	Previous name / location relative to P1 (kb)	Cell type expressing regulatory region RNA
R1RE15	human +62	Skin, Prostate, Liver, Breast, Brain, Lung, Haematopoietic, Ovary, Oesophagus, Colon, Mesenchymal, Kidney
R1RE16	mouse +59	Prostate, Lung, Haematopoietic, Mesenchymal
R1RE17	mouse +87	Haematopoietic, Mesenchymal
R1RE18	mouse +110 / human +139	Prostate, Haematopoiesis
R1RE19	mouse +171 / human +43	Skin, Prostate, Brain, Lung, Haematopoietic,Oesophagus, Mesenchymal
R1RE20	mouse +199	Liver, Haematopoietic,Colon
R1RE21	human intron 5.2 containing mouse +204	Skin, Prostate, Brain, Lung, Haematopoietic, Stomach, Colon, Mesenchymal, Kidney

Supplementary Table S2: SNP analysis of previously identified R1REs

Regulatory region	Number of SNPs (MAF>=1%)	SNP name	Predicted changes	SNP function
R1RE1	0	Not applicable	Not applicable	Not applicable
R1RE2	2	rs2834944	The T to C change alters 4 regulatory motifs.	Not investigated
		rs2834945	The T to C change is predicted to affect 11 regulatory motifs including GATA2 and PAX3 and PAX5.	Has an eQTL with oesophagus and is related to Gene expression of KMO in peripheral blood monocytes (p value: 1.36E-06) [1].
R1RE3	3	rs116951441	not predicted to change any motifs	Not investigated
		rs16993221	A to T change of rs16993221 alters 2 regulatory motifs BATF and IRF which work together in immune response.	Related to white blood cell count a critical marker which contributes to chronic inflammation (p value: 2E-8) [2].
		rs909143	The A to G change in rs909143 is predicted to affect 3 regulatory motifs	Has an eQTL with oesophagus and is related to gene expression of Kynurenine 3-monooxygenase gene in peripheral blood monocytes (p value: 1.84E-06) [1].
R1RE4	2	rs4817723	not predicted to change any motifs.	Not investigated
		rs12106380	T to G change of rs12106380 alters 6 regulatory motifs including GATA binding sites.	Not investigated
R1RE5	3	rs57911917	not predicted to change any motifs	Not investigated
		rs2834885	C to A change of rs2834885 alters 1 regulatory motif, PEBP.	Not investigated
		rs61607093	T to C change of rs61607093 alters 20 binding motifs including GATA3, Nanog, SPI1, Pax5 and p300	Not investigated
R1RE6	1	rs58035431	G to A change of alters 10 binding motifs including GATA2, p300	Not investigated
R1RE7	1	rs35526434	C to T change alters 12 regulatory motifs including SPI1, PAX5 and p300.	Not investigated

Regulatory region	Number of SNPs (MAF>=1%)	SNP name	Predicted changes	SNP function
R1RE8	3	rs189789980	T to G change alters 3 regulatory motifs.	Not investigated
		rs140039393	TTA to T change alters 4 binding motifs including Sox5.	Not investigated
		rs2834825	G to A change alters 19 motifs including Pou2f2.	Not investigated
R1RE9	0	Not applicable	Not applicable	Not applicable
R1RE10	13	rs2834768	G to A change of rs2834768 alters 13 regulatory motifs including MYC, TAL1 and multiple GATA sites.	Not investigated
		rs2834769	C to T alters 3 binding motifs.	Not investigated
		rs13049322	not predicted to change any motifs	Not investigated
		rs73374626	G to A change alters 5 motifs including MYC	Not investigated
		rs9984842	C to T is predicted to alter 12 motifs including KLF4, KLF7 and SP1.	Not investigated
		rs113221662	C to T has 7 predicted motif binding alterations including GATA3 and SP1.	Not investigated
		rs66840558	2 altered motifs.	Not investigated
		rs144641305	contains a change from AAG to A and is predicted to affect 10 binding sites including p300, PAX5, NANOG	Not investigated
		rs1883063	not predicted to change any motifs	Not investigated
		rs8129889	not predicted to change any motifs	Not investigated
		rs2834770	A to G change alters CEBPβ site and Hdx (brain related).	Not investigated

Regulatory region	Number of SNPs (MAF>=1%)	SNP name	Predicted changes	SNP function
		rs7283199	C to T change which predicts Sox17 (which modulates *WNT3A* and represses *RUNX1* expression) and HDAC2 motif changes	Has eQTLs reported with skin and thyroid
		rs41360844	G to C shows 2 predicted regulatory motif changes.	Not investigated
R1RE11	2	rs73902837	C to G predicted to change 2 regulatory regions	Not investigated
		rs2834756	T to C change alters 4 regulatory motifs	Not investigated
R1RE12	0	Not applicable	Not applicable	Not applicable
R1RE13	1	rs9978978	T to C change to change 1 regulatory motif	Not investigated
R1RE14	1	rs9976900	G to T changes 7 motifs	eQTL in the brain cortex [3] and was association with paediatric asthma and modulated by maternal smoking. [4].
R1RE15	2	rs933131	G to A change alters 4 regulatory motifs including Pax5 and Pax8.	Not investigated
		rs2834716	not predicted to alter any regulatory motifs	Not investigated
R1RE16	0	Not applicable	Not applicable	Not applicable
R1RE17	0	Not applicable	Not applicable	Not applicable
R1RE18	2	rs73900579	T to C change alters 2 regulatory motifs CEBPα and Pax4.	Has an eQTL with brain related to red cell distribution width [5].
		rs201708857	A to AG change is predicted to alter 20 regulatory motifs including CEBPA, SP1 and Pou2f2.	Not investigated
R1RE19	1	rs2284613	not predicted to alter any regulatory motifs	Not investigated

Regulatory region	Number of SNPs (MAF>=1%)	SNP name	Predicted changes	SNP function
R1RE20	0	Not applicable	Not applicable	Not applicable
R1RE21	3	rs2268276	(G major - A minor) alters 2 regulatory motifs.	These two SNPs (rs2268276 and Rs2249650) were found to be associated with acute myeloid leukaemia susceptibility. The different SNPs are in LD and change the ability of the enhancer. A (rs2249650) G (rs2268276) bases and AA have more enhancer capability that GA and GG. GA has the most reduced enhancer capability. The SNPs also affect SPI1 binding capability; AG has strong SPI1 binding, GA has weak SPI1 binding, whereas AA and GG have medium SPI1 binding capability [6]
		rs2249650	(G major- A minor) alters 4 binding motifs including CEBPA and Pouf2f.	
		rs8130772	A to G alters 5 binding motifs including SP1 and FLI1.	Not investigated

Supplementary Table S3: FATHMM prediction of the functional consequences of non-coding mutations of previously identified R1REs

Regulatory region	SNP name	Mutation	FATHMM Prediction	FATHMM Score
R1RE1	Not applicable	21,36399191,A,T	Pathogenic	0.92903
R1RE2	rs2834944	21,36854956,T,C	Neutral	0.17482
	rs2834945	21,36854998,T,C	Neutral	0.14062
R1RE3	rs116951441	21,36849201,C,T	Pathogenic	0.93214
	rs16993221	21,36849419,A,T	Neutral	0.14766
	rs909143	21,36849326,A,G	Neutral	0.14304
R1RE4	rs4817723	21,36801063,T,C	Neutral	0.14826
	rs12106380	21,36800304,T,G	Neutral	0.3271
R1RE5	rs57911917	21,36670442,C,T	Neutral	0.07334
	rs2834885	21,36670270,C,A	Neutral	0.04339
	rs61607093	21,36670200,T,C	Neutral	0.14469
R1RE6	rs58035431	21,36629289,G,A	Neutral	0.25715
R1RE7	rs35526434	21,36608572,C,T	Neutral	0.19116
R1RE8	rs189789980	21,36562426,T,G	Neutral	0.07144

Regulatory region	SNP name	Mutation	FATHMM Prediction	FATHMM Score
	rs140039393	21,36562422,TTA,T	Cannot be predicted by current software	Not applicable
	rs2834825	21,36562217,G,A	Neutral	0.15193
R1RE10	rs2834768	21,36478729,G,A	Neutral	0.05331
	rs2834769	21,36478973,C,T	Neutral	0.4585
	rs13049322	21,36479191,G,A	Neutral	0.17134
	rs73374626	21,36479570,G,A	Neutral	0.10596
	rs9984842	21,36479722,C,T	Neutral	0.10705
	rs113221662	21,36479732,C,T	Neutral	0.28179
	rs66840558	21,36479773,C,T	Neutral	0.114
	rs144641305	21,36479949,AAG,A	Cannot be predicted by current software	Not applicable
	rs1883063	21,36480002,C,T	Neutral	0.04077
	rs8129889	21,36480229,G,A	Neutral	0.08873
	rs2834770	21,36480487,A,G	Neutral	0.08439
	rs7283199	21,36481069,C,T	Neutral	0.09645
	rs41360844	21,36481471,G,C	Neutral	0.16723

**Regulatory region**	**SNP name**	**Mutation**	**FATHMM Prediction**	**FATHMM Score**
	rs199811665	21,36480715,C,A	Neutral	0.10044
	rs1440069314	21,36481470,C,T	Neutral	0.17852
	Not applicable	21,36480761,A,C	Neutral	0.08496
R1RE11	rs73902837	21,36464229,C,T	Neutral	0.25017
	rs2834756	21,36464234,T,C	Pathogenic	0.92189
R1RE13	rs9978978	21,36418601,T,C	Neutral	0.13753
R1RE14	rs9976900	21,36384363,G,T	Neutral	0.3077
R1RE15	rs933131	21,36359688,G,A	Neutral	0.29829
	rs2834716	21,36359392,G,A	Neutral	0.32485
	Not applicable	21,36359341,T,G	Cannot be predicted by current software	Not applicable
R1RE18	rs73900579	21,36280897,T,C	Pathogenic	0.93519
	rs201708857	21,36280873,A,AG	Cannot be predicted by current software	Not applicable
	Not applicable	21,36280880,A,G	Cannot be predicted by current software	Not applicable
R1RE19	rs2284613	21,36218264,A,G	Pathogenic	0.88633
R1RE21	rs2268276	21,36181014,G,A	Pathogenic	0.87621

Regulatory region	SNP name	Mutation	FATHMM Prediction	FATHMM Score
	rs2249650	21,36180986,A,G	Pathogenic	0.87184
	rs8130772	21,36180684,A,G	Neutral	0.32349
	Not applicable	21,36180724,G,A	Cannot be predicted by current software	Not applicable

References

1. Zeller, T., et al., *Genetics and Beyond – The Transcriptome of Human Monocytes and Disease Susceptibility.* PLOS ONE, 2010. **5**(5): p. e10693.
2. Kong, M. and C. Lee, *Genetic associations with C-reactive protein level and white blood cell count in the KARE study.* International Journal of Immunogenetics, 2013. **40**(2): p. 120-125.
3. Heinzen, E.L., et al., *Tissue-specific genetic control of splicing: implications for the study of complex traits.* PLoS Biol, 2008. **6**(12): p. e1000001.
4. Haley, K.J., et al., *RUNX transcription factors: association with pediatric asthma and modulated by maternal smoking.* American Journal of Physiology-Lung Cellular and Molecular Physiology, 2011. **301**(5): p. L693-L701.
5. Kichaev, G., et al., *Leveraging Polygenic Functional Enrichment to Improve GWAS Power.* The American Journal of Human Genetics, 2019. **104**(1): p. 65-75.
6. Xu, X., et al., *Identification and functional analysis of acute myeloid leukemia susceptibility associated single nucleotide polymorphisms at non-protein coding regions of RUNX1.* Leukemia & lymphoma, 2016. **57**(6): p. 1442-1449.
